# (*E*)-4-Fluoro-2-[(4-hy­droxy­pheneth­yl)imino­meth­yl]phenol

**DOI:** 10.1107/S1600536811055875

**Published:** 2012-01-11

**Authors:** Rui-Qin Fang, Tao Song, Yu-Xiang Li

**Affiliations:** a School of Life Science and Technology, University of Electronic Science and Technology of China, Chengdu 610054, People’s Republic of China; b State Key Laboratory of Pharmaceutical Biotechnology, Nanjing University, Nanjing 210093, People’s Republic of China

## Abstract

The title compound, C_15_H_14_FNO_2_, has an *E* conformation about the C=N bond, which facilitates the formation of an intra­molecular O—H⋯N hydrogen bond. The F atom is disordered over two adjacent sites in a 0.65 (7):0.35 (7) ratio. The dihedral angle between the benzene ring planes is 14.2 (2)°. In the crystal, mol­ecules are linked by O—H⋯O hydrogen bonds, forming *C*(14) [010] chains.

## Related literature

For a related structure, see: Li *et al.* (2006 ▶). For reference bond lengths, see: Allen *et al.* (1987 ▶).
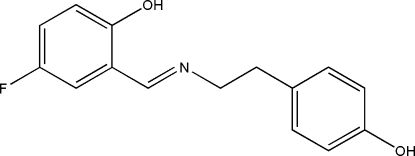


## Experimental

### 

#### Crystal data

C_15_H_14_FNO_2_
*M* *_r_* = 259.27
Monoclinic, 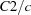

*a* = 15.979 (3) Å
*b* = 12.941 (3) Å
*c* = 15.040 (3) Å
β = 121.72 (3)°
*V* = 2645.5 (9) Å^3^
*Z* = 8
Mo *K*α radiation
μ = 0.10 mm^−1^
*T* = 293 K
0.40 × 0.30 × 0.30 mm

#### Data collection

Enraf–Nonius CAD-4 diffractometer
Absorption correction: ψ scan (North *et al.*, 1968 ▶) *T*_min_ = 0.963, *T*_max_ = 0.972
2692 measured reflections
2599 independent reflections
1431 reflections with *I* > 2σ(*I*)
*R*_int_ = 0.052
3 standard reflections every 200 reflections intensity decay: 1%

#### Refinement

*R*[*F* ^2^ > 2σ(*F* ^2^)] = 0.064
*wR*(*F* ^2^) = 0.179
*S* = 1.03
2599 reflections
185 parameters
H-atom parameters constrained
Δρ_max_ = 0.31 e Å^−3^
Δρ_min_ = −0.21 e Å^−3^

### 

Data collection: *CAD-4 Software* (Enraf–Nonius, 1989 ▶); cell refinement: *CAD-4 Software*; data reduction: *XCAD4* (Harms & Wocadlo, 1995 ▶); program(s) used to solve structure: *SHELXS97* (Sheldrick, 2008 ▶); program(s) used to refine structure: *SHELXL97* (Sheldrick, 2008 ▶); molecular graphics: *SHELXTL* (Sheldrick, 2008 ▶); software used to prepare material for publication: *SHELXL97*.

## Supplementary Material

Crystal structure: contains datablock(s) global, I. DOI: 10.1107/S1600536811055875/hb6581sup1.cif

Structure factors: contains datablock(s) I. DOI: 10.1107/S1600536811055875/hb6581Isup2.hkl

Supplementary material file. DOI: 10.1107/S1600536811055875/hb6581Isup3.cdx

Supplementary material file. DOI: 10.1107/S1600536811055875/hb6581Isup4.cml

Additional supplementary materials:  crystallographic information; 3D view; checkCIF report

## Figures and Tables

**Table 1:** Hydrogen-bond geometry (Å, °)

*D*—H⋯*A*	*D*—H	H⋯*A*	*D*⋯*A*	*D*—H⋯*A*
O1—H1⋯N1	0.82	1.82	2.565 (3)	150
O2—H2⋯O1^i^	0.82	1.89	2.707 (3)	173

Symmetry code: (i) 
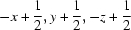
.

**Fig. 1.**
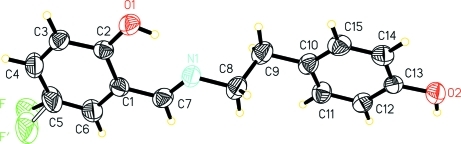
The structure of the title compound (I) showing 35% probability displacement ellipsoids.

**Fig. 2.**
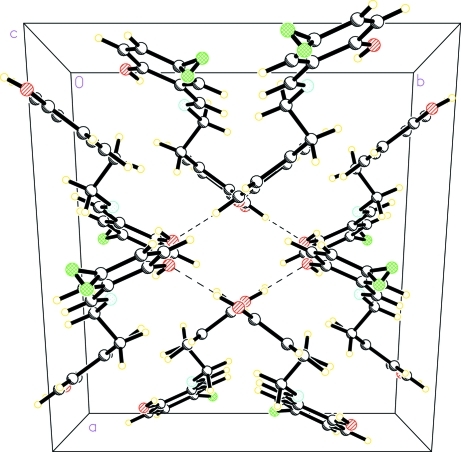
The crystal packing of (I), viewed along the b axis. Hydrogen bonds are shown as dashed lines.

## supplementary crystallographic information

### Comment

The cystal structure of (*E*)-2-((4-hydroxyphenethylimino)methyl)phenol, synthesized by salicylaldehyde and tyramine, has been reported before (Li *et al.*, 2006). There are no fluoro subsitituent on the 5-position of benzene, as compared with the title compound. The molecular structure of title compound (I), Fig. 1, possesses an E configuration about C7=N1 double bond, and the bond length 1.283 (3) Å is in the normal range (Allen *et al.*, 1987). Disorder is observed concerning fluoro atom in a ratio of 0.35 (7): 0.65 (7). The torsion angle of C9—C8—N1—C7 and N1—C8—C9—C10 are 123.3 (3) ° and 177.8 (2) °, respectively. The dihedral angles between two benzene ring planes is 14.18 (20) °. In the crystal, intramolecular O1—H1···N1 hydrogen bonds occur, and the intermolecular O2—H2···O1 hydrogen bonds lead to chains of molecules along the *b* axis.(Fig. 2).

### Experimental

The title compound was prepared by stirring a mixture of 5-fluoro-salicylaldehyde (122 mg, 1 mmol) and tyramine (137 mg, 1 mmol) in methanol (15 ml) for 2 h at room temperature. After keeping the solution in air for 3 d, brown block-shaped crystals of (I) were formed. The crystals were isolated, washed three times with methanol and dried in a vacuum desiccator containing anhydrous CaCl_2_.

### Refinement

Disorder is observed concerning F [0.35 (7)] and F'[0.65 (7)]. All the H atoms, were placed in idealized positions (C—H = 0.93- 0.96 Å, O—H = 0.82 Å) and refined as riding with *U*_iso_(H) = 1.2*U*_eq_(C) and *U*_iso_(H) = 1.5*U*_eq_(O).

### Crystal data

**Table:**

C_15_H_14_FNO_2_	*F*(000) = 1088
*M**_r_* = 259.27	*D*_x_ = 1.302 Mg m^−^^3^
Monoclinic, *C*2/*c*	Mo *K*α radiation, λ = 0.71073 Å
Hall symbol: -C 2yc	Cell parameters from 1056 reflections
*a* = 15.979 (3) Å	θ = 3.2–23.5°
*b* = 12.941 (3) Å	µ = 0.10 mm^−^^1^
*c* = 15.040 (3) Å	*T* = 293 K
β = 121.72 (3)°	Block, brown
*V* = 2645.5 (9) Å^3^	0.40 × 0.30 × 0.30 mm
*Z* = 8	

### Data collection

**Table:**

Enraf–Nonius CAD-4 diffractometer	1431 reflections with *I* > 2σ(*I*)
Radiation source: fine-focus sealed tube	*R*_int_ = 0.052
graphite	θ_max_ = 26.0°, θ_min_ = 2.2°
ω/2θ scan	*h* = 0→19
Absorption correction: ψ scan (North *et al.*, 1968)	*k* = 0→15
*T*_min_ = 0.963, *T*_max_ = 0.972	*l* = −18→15
2692 measured reflections	3 standard reflections every 200 reflections
2599 independent reflections	intensity decay: 1%

### Refinement

**Table:**

Refinement on *F*^2^	Secondary atom site location: difference Fourier map
Least-squares matrix: full	Hydrogen site location: inferred from neighbouring sites
*R*[*F*^2^ > 2σ(*F*^2^)] = 0.064	H-atom parameters constrained
*wR*(*F*^2^) = 0.179	*w* = 1/[σ^2^(*F*_o_^2^) + (0.0762*P*)^2^ + 1.0201*P*] where *P* = (*F*_o_^2^ + 2*F*_c_^2^)/3
*S* = 1.03	(Δ/σ)_max_ < 0.001
2599 reflections	Δρ_max_ = 0.31 e Å^−^^3^
185 parameters	Δρ_min_ = −0.20 e Å^−^^3^
0 restraints	Extinction correction: *SHELXL97* (Sheldrick, 2008), Fc^*^=kFc[1+0.001xFc^2^λ^3^/sin(2θ)]^-1/4^
Primary atom site location: structure-invariant direct methods	Extinction coefficient: 0.0055 (9)

### Special details

**Table:**

Geometry. All e.s.d.'s (except the e.s.d. in the dihedral angle between two l.s. planes) are estimated using the full covariance matrix. The cell e.s.d.'s are taken into account individually in the estimation of e.s.d.'s in distances, angles and torsion angles; correlations between e.s.d.'s in cell parameters are only used when they are defined by crystal symmetry. An approximate (isotropic) treatment of cell e.s.d.'s is used for estimating e.s.d.'s involving l.s. planes.
Refinement. Refinement of *F*^2^ against ALL reflections. The weighted *R*-factor *wR* and goodness of fit *S* are based on *F*^2^, conventional *R*-factors *R* are based on *F*, with *F* set to zero for negative *F*^2^. The threshold expression of *F*^2^ > σ(*F*^2^) is used only for calculating *R*-factors(gt) *etc*. and is not relevant to the choice of reflections for refinement. *R*-factors based on *F*^2^ are statistically about twice as large as those based on *F*, and *R*- factors based on ALL data will be even larger.

### Fractional atomic coordinates and isotropic or equivalent isotropic displacement parameters (Å^2^)

**Table:**

	*x*	*y*	*z*	*U*_iso_*/*U*_eq_	Occ. (<1)
C1	0.42192 (19)	0.8367 (2)	0.0160 (2)	0.0580 (7)	
C2	0.46488 (18)	0.7365 (2)	0.0453 (2)	0.0534 (7)	
C3	0.5012 (2)	0.6926 (2)	−0.0131 (2)	0.0610 (7)	
H3	0.5303	0.6276	0.0050	0.073*	
C4	0.4948 (2)	0.7430 (3)	−0.0953 (3)	0.0810 (10)	
H4	0.5193	0.7128	−0.1333	0.097*	
C5	0.4516 (3)	0.8397 (3)	−0.1224 (3)	0.1024 (13)	
C6	0.4166 (3)	0.8873 (3)	−0.0690 (3)	0.0888 (11)	
H6	0.3893	0.9530	−0.0884	0.107*	
C7	0.38330 (19)	0.8852 (2)	0.0720 (2)	0.0642 (8)	
H7	0.3559	0.9508	0.0515	0.077*	
C8	0.3470 (2)	0.8918 (2)	0.2089 (2)	0.0725 (9)	
H8A	0.3195	0.9586	0.1782	0.087*	
H8B	0.4007	0.9030	0.2802	0.087*	
C9	0.2695 (2)	0.8273 (2)	0.2094 (3)	0.0762 (9)	
H9A	0.2963	0.7595	0.2375	0.091*	
H9B	0.2147	0.8184	0.1382	0.091*	
C10	0.23315 (19)	0.8764 (2)	0.2740 (2)	0.0637 (8)	
C11	0.1726 (2)	0.9632 (2)	0.2386 (2)	0.0662 (8)	
H11	0.1546	0.9911	0.1739	0.079*	
C12	0.13856 (19)	1.0090 (2)	0.2964 (2)	0.0604 (7)	
H12	0.0985	1.0671	0.2709	0.072*	
C13	0.1641 (2)	0.9681 (2)	0.3921 (2)	0.0581 (7)	
C14	0.2249 (2)	0.8830 (2)	0.4294 (2)	0.0709 (8)	
H14	0.2434	0.8556	0.4944	0.085*	
C15	0.2583 (2)	0.8387 (2)	0.3706 (2)	0.0709 (8)	
H15	0.2993	0.7813	0.3969	0.085*	
F	0.419 (2)	0.865 (3)	−0.230 (2)	0.109 (7)	0.35 (7)
F'	0.462 (4)	0.900 (3)	−0.191 (4)	0.155 (10)	0.65 (7)
N1	0.38481 (16)	0.84202 (18)	0.14957 (18)	0.0634 (7)	
O1	0.46969 (16)	0.68714 (14)	0.12319 (15)	0.0701 (6)	
H1	0.4446	0.7223	0.1484	0.105*	
O2	0.13257 (18)	1.00897 (15)	0.45299 (17)	0.0813 (7)	
H2	0.1042	1.0638	0.4277	0.122*	

### Atomic displacement parameters (Å^2^)

**Table:**

	*U*^11^	*U*^22^	*U*^33^	*U*^12^	*U*^13^	*U*^23^
C1	0.0563 (16)	0.0548 (16)	0.0674 (17)	0.0037 (13)	0.0356 (14)	0.0071 (13)
C2	0.0498 (15)	0.0504 (16)	0.0570 (16)	−0.0013 (12)	0.0261 (13)	0.0013 (12)
C3	0.0576 (16)	0.0568 (17)	0.0675 (18)	0.0049 (13)	0.0320 (15)	−0.0015 (14)
C4	0.084 (2)	0.091 (2)	0.093 (2)	0.0150 (19)	0.064 (2)	0.008 (2)
C5	0.135 (3)	0.101 (3)	0.119 (3)	0.036 (2)	0.099 (3)	0.048 (2)
C6	0.112 (3)	0.071 (2)	0.117 (3)	0.0284 (19)	0.083 (2)	0.033 (2)
C7	0.0601 (17)	0.0536 (16)	0.080 (2)	0.0072 (13)	0.0379 (16)	0.0005 (14)
C8	0.0685 (19)	0.073 (2)	0.081 (2)	0.0003 (15)	0.0427 (17)	−0.0167 (16)
C9	0.0679 (19)	0.074 (2)	0.092 (2)	0.0005 (16)	0.0456 (18)	−0.0165 (17)
C10	0.0554 (16)	0.0629 (18)	0.076 (2)	0.0038 (14)	0.0367 (15)	−0.0064 (15)
C11	0.0640 (17)	0.0708 (19)	0.0629 (18)	0.0087 (15)	0.0328 (15)	0.0015 (14)
C12	0.0542 (16)	0.0575 (17)	0.0662 (18)	0.0104 (12)	0.0294 (14)	0.0029 (14)
C13	0.0636 (16)	0.0485 (15)	0.0713 (18)	−0.0033 (13)	0.0418 (15)	−0.0040 (13)
C14	0.084 (2)	0.0572 (17)	0.077 (2)	0.0102 (16)	0.0461 (18)	0.0121 (15)
C15	0.0696 (19)	0.0562 (18)	0.086 (2)	0.0164 (14)	0.0405 (17)	0.0111 (16)
F	0.158 (13)	0.103 (10)	0.095 (11)	0.016 (9)	0.088 (12)	0.038 (6)
F'	0.25 (2)	0.134 (10)	0.186 (16)	0.067 (13)	0.182 (17)	0.072 (12)
N1	0.0639 (14)	0.0665 (15)	0.0672 (15)	0.0008 (11)	0.0394 (13)	−0.0078 (12)
O1	0.0915 (15)	0.0562 (12)	0.0742 (13)	0.0158 (10)	0.0515 (12)	0.0103 (10)
O2	0.1126 (18)	0.0673 (14)	0.0924 (15)	0.0134 (12)	0.0734 (15)	0.0073 (11)

### Geometric parameters (Å, °)

**Table:**

C1—C6	1.399 (4)	C8—H8B	0.9700
C1—C7	1.425 (4)	C9—C10	1.511 (4)
C1—C2	1.425 (4)	C9—H9A	0.9700
C2—O1	1.300 (3)	C9—H9B	0.9700
C2—C3	1.403 (3)	C10—C15	1.376 (4)
C3—C4	1.353 (4)	C10—C11	1.392 (4)
C3—H3	0.9300	C11—C12	1.379 (4)
C4—C5	1.383 (4)	C11—H11	0.9300
C4—H4	0.9300	C12—C13	1.378 (4)
C5—C6	1.345 (4)	C12—H12	0.9300
C5—F'	1.373 (11)	C13—O2	1.363 (3)
C5—F	1.456 (18)	C13—C14	1.378 (4)
C6—H6	0.9300	C14—C15	1.376 (4)
C7—N1	1.283 (3)	C14—H14	0.9300
C7—H7	0.9300	C15—H15	0.9300
C8—N1	1.464 (3)	O1—H1	0.8200
C8—C9	1.496 (4)	O2—H2	0.8200
C8—H8A	0.9700		
			
C6—C1—C7	119.8 (3)	H8A—C8—H8B	108.0
C6—C1—C2	119.8 (3)	C8—C9—C10	111.6 (2)
C7—C1—C2	120.4 (2)	C8—C9—H9A	109.3
O1—C2—C3	121.2 (2)	C10—C9—H9A	109.3
O1—C2—C1	121.1 (2)	C8—C9—H9B	109.3
C3—C2—C1	117.7 (2)	C10—C9—H9B	109.3
C4—C3—C2	121.4 (3)	H9A—C9—H9B	108.0
C4—C3—H3	119.3	C15—C10—C11	116.7 (3)
C2—C3—H3	119.3	C15—C10—C9	122.2 (3)
C3—C4—C5	119.4 (3)	C11—C10—C9	121.2 (3)
C3—C4—H4	120.3	C12—C11—C10	122.0 (3)
C5—C4—H4	120.3	C12—C11—H11	119.0
C6—C5—F'	115.9 (10)	C10—C11—H11	119.0
C6—C5—C4	122.6 (3)	C13—C12—C11	119.7 (3)
F'—C5—C4	119.9 (5)	C13—C12—H12	120.1
C6—C5—F	123.0 (9)	C11—C12—H12	120.1
F'—C5—F	32.0 (12)	O2—C13—C14	117.8 (3)
C4—C5—F	111.2 (12)	O2—C13—C12	122.8 (2)
C5—C6—C1	119.1 (3)	C14—C13—C12	119.3 (3)
C5—C6—H6	120.4	C15—C14—C13	120.0 (3)
C1—C6—H6	120.4	C15—C14—H14	120.0
N1—C7—C1	122.6 (3)	C13—C14—H14	120.0
N1—C7—H7	118.7	C14—C15—C10	122.2 (3)
C1—C7—H7	118.7	C14—C15—H15	118.9
N1—C8—C9	111.4 (2)	C10—C15—H15	118.9
N1—C8—H8A	109.3	C7—N1—C8	123.1 (2)
C9—C8—H8A	109.3	C2—O1—H1	109.5
N1—C8—H8B	109.3	C13—O2—H2	109.5
C9—C8—H8B	109.3		
			
C6—C1—C2—O1	−179.2 (3)	C2—C1—C7—N1	−0.3 (4)
C7—C1—C2—O1	0.1 (4)	N1—C8—C9—C10	177.8 (2)
C6—C1—C2—C3	0.5 (4)	C8—C9—C10—C15	−106.5 (3)
C7—C1—C2—C3	179.7 (2)	C8—C9—C10—C11	73.0 (3)
O1—C2—C3—C4	178.8 (3)	C15—C10—C11—C12	−0.5 (4)
C1—C2—C3—C4	−0.8 (4)	C9—C10—C11—C12	179.9 (3)
C2—C3—C4—C5	0.0 (5)	C10—C11—C12—C13	−0.4 (4)
C3—C4—C5—C6	1.2 (6)	C11—C12—C13—O2	−179.5 (2)
C3—C4—C5—F'	167 (3)	C11—C12—C13—C14	1.2 (4)
C3—C4—C5—F	−159.1 (16)	O2—C13—C14—C15	179.7 (3)
F'—C5—C6—C1	−167 (3)	C12—C13—C14—C15	−1.0 (4)
C4—C5—C6—C1	−1.5 (6)	C13—C14—C15—C10	0.1 (5)
F—C5—C6—C1	156 (2)	C11—C10—C15—C14	0.7 (4)
C7—C1—C6—C5	−178.6 (3)	C9—C10—C15—C14	−179.7 (3)
C2—C1—C6—C5	0.6 (5)	C1—C7—N1—C8	178.9 (2)
C6—C1—C7—N1	179.0 (3)	C9—C8—N1—C7	123.3 (3)

### Hydrogen-bond geometry (Å, °)

**Table:**

*D*—H···*A*	*D*—H	H···*A*	*D*···*A*	*D*—H···*A*
O1—H1···N1	0.82	1.82	2.565 (3)	150
O2—H2···O1^i^	0.82	1.89	2.707 (3)	173

Symmetry codes: (i) −*x*+1/2, *y*+1/2, −*z*+1/2.
